# Kinetics of 5% and 20% albumin: A controlled crossover trial in volunteers

**DOI:** 10.1111/aas.14074

**Published:** 2022-05-13

**Authors:** Markus Zdolsek, Robert G. Hahn

**Affiliations:** ^1^ Department of Biomedical and Clinical Sciences (BKV) Linköping University Linköping Sweden; ^2^ Research Unit Södertälje Hospital, Södertälje, Sweden, and Karolinska Institutet at Danderyds Hospital (KIDS) Danderyd Sweden

**Keywords:** albumin, analysis; serum albumin, human, hyperoncotic; capillary permeability, pharmacokinetics, physiology, plasma albumin, plasma volume, volume therapy; physiology

## Abstract

**Background:**

Albumin for intravenous infusion is marketed in two concentrations, 20% and 5%, but how they compare with regard to plasma volume expansion over time is unclear.

**Methods:**

In a prospective crossover study, 12 volunteers received 3 ml kg^−1^ of 20% albumin and, on another occasion, 12 ml kg^−1^ of 5% albumin over 30 min. Hence, equivalent amounts of albumin were given. Blood was collected on 15 occasions over 6 h. Mass balance and volume kinetics were used to estimate the plasma volume expansion and the capillary leakage of albumin and fluid based on measurements of blood hemoglobin, plasma albumin, and the colloid osmotic pressure.

**Results:**

The greatest plasma volume expansion was 16.0 ± 6.4% (mean ± *SD*) with 20% albumin and 19.0 ± 5.2% with 5% albumin (*p* < .03). The volume expansion with 20% albumin corresponded to twice the infused volume. One third of the 5% albumin volume quickly leaked out of the plasma, probably because of the higher colloid osmotic pressure of the volunteer plasma (mean, 24.5 mmHg) than the albumin solution (19.1 mmHg). At 6 h, the capillary leakage amounted to 42 ± 15% and 47 ± 11% of the administered albumin with the 20% and 5% preparations, respectively (*p* = .28). The corresponding urine outputs were 547 (316–780) ml and 687 (626–1080) ml (median and interquartile range; *p* = .24).

**Conclusion:**

The most important difference between the fluids was a dehydrating effect of 20% albumin when the same albumin mass was administered.

**Editorial Comment**

In this prospective cross over kinetic study, volunteers received equivalent amount of albumin, either 5% or 20%. Five per cent albumin infusion resulted in larger plasma volume expansion and urinary excretion than 20% albumin during the first hour. Both preparations provided long‐lasting plasma volume expansion. A clinically possibly important difference is a dehydrating effect of 20% albumin.

## 1 INTRODUCTION

Albumin for intravenous infusion has been used for plasma volume (PV) expansion for the past 75 years. The short‐term PV expansion is greater with albumin than with isotonic crystalloid fluids due to the colloid osmotic properties of the albumin molecule.^1^, ^2^ Fluid resuscitation with albumin as compared with crystalloids might have beneficial effects on hemodynamics and outcomes of surgery and intensive care.^2^, ^3^ Synthetic colloids have been largely abandoned and albumin has then become the colloid of choice for many clinicians.

Both hyper‐oncotic (20%–25%) and slightly hypo‐oncotic (4%–5%) albumin preparations are marketed. The 20% solution is an attractive option if the clinician wants to reduce the administered fluid volume.^4^ Larger volumes of 5% albumin than 20% albumin are usually infused as PV expansion due to the difference in concentration. Therefore, 5% albumin is more likely to be followed by a positive fluid balance and edema, which increase the risk of anastomosis insufficiency,^5^ paralytic ileus,^6^ and impaired wound healing.^7^

Previous studies show that 20% albumin expands the PV by twice the infused volume rather than by the four times suggested by the excess albumin concentration.^8^, ^9^ The larger volumes of 5% albumin given may induce a larger diuresis, both by improving the renal vascular perfusion and by more strongly stimulating the release of atrial natriuretic peptide (ANP) from the heart. However, the degree to which these preparations can be interchanged is a frequent clinical issue that is still unclear, in particular since the two solutions are commonly administered in very different volumes.

The purpose of the present study was to compare the PV expansion, the strength of the oncotic‐driven recruitment of extravascular fluid, and the intravascular half‐life of 20% and 5% albumin when infused in healthy volunteers. Equal albumin masses were administered over 30 min. The primary end point was the difference in fluid balance between the two albumin preparations at 6 h. The null hypothesis was that capillary leakage and urinary excretion would equilibrate the difference in the infused volume.

## 2 METHODS

### 2.1 Approvals

This prospective one‐center, open‐controlled trial compared the pharmacological characteristics of 20% and 5% albumin. The Regional Ethics Committee of Linköping (Dnr 2017/478‐31) and the Swedish Medical Products Agency approved the study, which was registered as Eudra‐CT 2017‐003687‐12 before any subject was enrolled. All participants gave their informed consent orally and in writing. No data from this trial have previously been published. A third arm comprised a slower infusion of 20% albumin but will be reported elsewhere. The presentation follows the CONSORT checklist.

### 2.2 Participants

Twelve volunteers, six males and six females, were recruited through posters at Linköping University Hospital. Inclusion criteria included age between 18 and 60 years, absence of medical disease, and no daily medication. Exclusion criteria were pregnancy and severe allergy.

### 2.3 Intervention

Participants arrived at the study location at Linköping University Hospital at 7 a.m. Food and water had been restricted from midnight and included only the intake of one sandwich and 200 ml of liquid 1.5 h before arriving at the hospital.

The volunteers received one venous cannula in each arm. They voided voluntarily 30 min prior to the initiation of the study and then rested in the supine position. Baseline samples were drawn through one of the cannulas. In the other cannula, an infusion of 3 ml kg^−1^ of 20% albumin was administered. On a second occasion at least 3 weeks from this first infusion, 12 ml kg^−1^ of 5% albumin was administered. Both fluids were given at a constant rate over 30 min via an infusion pump. No other fluid was administered during the experiment.

Blood was withdrawn for analysis at 15 exactly timed occasions over a period of 6 h after starting the infusion (0, 10, 20, 30, 40, 50, 60, 75, 90, 120, 150, 180, 240, 300, and 360 min). The volunteers were allowed to void in bedpans whenever needed, and the urine volume was measured. Urine was analyzed at baseline, 1 h, and 6 h.

The arterial pressure and the heart rate were measured by an automatic cuff at 0, 1, 2.5, and 6 h (Dräger Infinity M540). Monitoring also included pulse oximetry.

### 2.4 Blood, urine, and fluid analyses

Whole blood was analyzed for hemoglobin (Hb) concentration and hematocrit (Hct) with a coefficient of variation (CV) of 0.9%, as given by duplicate samples at baseline, on a Cell‐Dyn Sapphire (Abbott Diagnostics).

Plasma was analyzed for albumin, creatinine, sodium, and potassium on a Cobas 8000 (Roche) with CV of 2.3%, 1.9%, 0.7%, and 1.0%.

The colloid osmotic pressures (COP) of the infused fluids and the sampled plasma were measured on an Osmomat 050 (Gonotec) with a CV of 2%.

The plasma concentration of mid‐regional pro‐atrial natriuretic peptide (MR‐proANP) at baseline and 30 min after the infusion ended was measured by radioimmunoassay (Brahms MR‐proANP Kryptor) with a CV of <3.5%. The manufacturer reports that the median value in healthy humans is 46 pmol l^−1^. Urine was analyzed for creatinine with a CV of 1.8% on the Cobas 8000.

### 2.5 Mass balance calculations

The mass balance and volume kinetic calculations summarized below are explained in detail in Supplementary Digital File 1.

The changes in blood volume during the experiment were calculated by multiplying the hemodilution (Hb_baseline_/Hb) after making a correction for the sampled blood volume (total 156 ml),^10^ with the baseline blood volume, which was estimated based on the height and weight of each volunteer.^11^ Conversion from blood volume to PV was done by multiplication with the measured baseline hematocrit (Hct).

The fractions of the infused fluid volume that was retained in the PV was obtained as the product of PV at baseline (in ml) and the relative increase of the PV (PV/PV_baseline_) divided by the infused volume.

The albumin mass was taken as the product of the PV and the plasma albumin concentration.

The capillary leakage of albumin was obtained as the change in albumin mass, with correction for the infused amount of albumin, between baseline and any later time.

The half‐life of the infused albumin was obtained from the logarithm of the slope of the albumin mass versus time.^8^, ^9^ The half‐life of the decay of the PV expansion was estimated in the same way for the albumin mass.

### 2.6 Kinetic models

Two models were used to analyze the kinetics of the excess albumin mass and the excess fluid provided by the infusions. The first was a one‐compartment model that captured the distribution volume (*V*_c_) and distribution rate constant (*k*_b_) for the infused albumin molecules. The dependent variable was the plasma albumin concentration multiplied by the relative change of the PV, which was given by PV/PV_baseline_.

The second model analyzed the kinetics of the infused fluid volume and consisted of one absorption route and two elimination routes.^12^ Absorption of interstitial fluid to the plasma occurred at a rate proportional by a rate constant *k*_21_ to the interstitial fluid volume (IFV), which amounts to 15% of the body weight.^13^

One elimination route was the urinary excretion (*k*_10_) and the second was the capillary leakage of fluid (*k*_b_). The functional PV at baseline was termed *V*_c_ and the expanded volume *v*_c._ The model equations are:

$$dv_{c}/dt=R_{o}–k_{b}\;\left(v_{c}–V_{c}\right)–k_{10}\;\left(v_{c}–V_{c}\right)+k_{21}\;\mathrm{IFV}$$

$$\text{dIFV}/dt=-k_{21}\;\mathrm{IFV}$$

$$du/dt=k_{10}\;\left(v_{c}–V_{c}\right)$$

where *R*_o_ is the rate of infusion and *u* is the measured urinary excretion. Note that the rate constant *k*_21_ is not operational before an infusion begins.

The dependent variables were the frequently measured plasma dilution and the urinary excretion measured at 1 and 6 h. The plasma dilution at any time was given by [(PV/PV_baseline_) – 1]. This dilution equals [(*v*_c_ – *V*_c_)/*V*_c_] in the model, while (*v*_c_ – *V*_c_) denotes the central volume expansion.

The fixed parameters in the albumin model (*V*_c_ and *k*_b_) were estimated simultaneously for all 24 experiments using the First Order Conditional Estimation Extended Least‐Squares (FOCE ELS) search routine in the Phoenix software for nonlinear mixed effects (NLME), version 8.2 (Pharsight, St. Louis, MO), and the additive model for the within‐subject variability. All parameters in the fluid model (*k*_21,_ *V*_c_, *k*_10_, *k*_b_) were also estimated in a single run.

### 2.7 Covariate analysis

The kinetic models described in Section 2.6 were refined by adding individual‐specific *covariates*, as guided by so‐called “eta plots.” The use of covariates improves the precision of a kinetic model by considering variables that vary between subjects, such as age and gender.^14^ In the present study, 10 potential covariates were considered and tested against all parameters in the basic kinetic models. Age, gender, body weight, height, body mass index, and the baseline concentrations of Hb and urine creatinine were entered once for each patient. Plasma creatinine and plasma MR‐proANP were measured twice per experiment and applied at the point in time when measured. COP was entered 15 times per experiment at the same points in time as Hb and plasma albumin.

The osmotic redistribution of body fluid was considered by entering the difference between the COP of the infused solution and the plasma COP as at each point in time during the infusion period, while 0 was entered for each point in time post‐infusion.

The power and the linear covariate models were applied in the present study. Both are used for continuous data but only the linear model accepts zero and negative values. In a power model, analysis of all subjects yields a “typical value” (tv) of the parameter for the group which is then varied according to an exponent in proportion to the deviation between the variable value in the individual and the mean value for the group.

An example is how the urinary excretion predicted by the model is modified by the urine creatinine concentration at baseline, which depends on the habitual ingestion of water.^15^ Here, the tv*k*_10_ is 3.41 × 10^−3^ min^−1^, the covariate exponent − 0.49, and the mean concentration 15.6 mmol/L. For a subject with urine creatinine at the mean of the highest quartile (25 mmol/L), the value of *k*_10_ becomes modified to 3.41 × 10^−3^ × [(25/15.6)^–0.49^], which is 21% lower than mean urine flow rate for the group. The mean of the lowest quartile (7.3 mmol/L) yields a urine flow rate being 45% higher than the mean for the group.

### 2.8 Outcome measures

The primary outcome measure was the difference in fluid balance between the two albumin preparations at 6 h. Secondary outcomes were the maximum PV expansion, differences in extravascular fluid recruitment, COP, and the intravascular half‐lives of albumin and fluid.

### 2.9 Statistics

Data were reported as the mean ± standard deviation (*SD*). Differences between the two infusions were evaluated by using the paired *t* test.

If the data showed a skewed distribution, the results were given as the median (25th–75th percentiles), and group differences were studied with the Wilcoxon matched‐pair test. *p* < .05 was statistically significant.

Power analysis was based on the data for the plasma expansion of 15.8 ± 4.9% at the end of an infusion of 3 ml kg^−1^ of 20% albumin over 30 min.^11^ A difference in PV expansion between the two groups amounting to 20% of that mean value (effect size 0.64) yielded 22 experiments at the *p* < .05 level and with a certainty of 80%.

Kinetic parameters were given as the best estimate and 95% confidence interval (CI). The “best estimate” is the population value that that corresponds to the lowest −2 log likelihood (−2 LL) for the model's objective function. A new covariate was accepted if its 95% CI did not include 0, if its inclusion decreased the −2 LL for the model by >6.6 points (*p* < .01), and the identification could be confirmed by both forward and backward elimination. The power model was chosen for covariates that consisted of continuous data >0 and the linear model for data that included 0 and/or negative values.

The goodness‐of‐fit of the final model was evaluated with *residual plots*, where each model‐predicted value is compared with the measured values.

The performance of the final model was illustrated with *predictive checks*, which compares the confidence limits of the computer‐generated changes in plasma albumin (including covariates) with the confidence limits for the measured albumin‐time curves.

The goodness‐of‐fit of the basic model (without covariates) was examined by calculating the Conditional Weighted Residuals (CWRES), which is the difference between model‐predicted and measured data divided by the root of the covariance.^16^ This residual is appropriate the use when a FOCE search routine is used. Good model specification is supported by an even distribution of residuals around zero and few data points >±3 *SD*s.

## 3 RESULTS

### 3.1 Basic data

The 24 experiments were performed between February 2018 and December 2018.

The volunteers were 28 ± 10 years old, weighted 72 ± 12 kg, and had a BMI of 24.1 ± 2.7 kg/m^2^. The mean arterial pressure was 92 ± 10 mmHg and the heart rate was 64 ± 9 bpm just before the infusions started.

The volume of infused albumin amounted to 219 ± 33 ml for the 20% solution and 877 ± 132 ml for the 5% solution. Details about the fluid therapy are presented in Table 1. All individual data are given in Supplementary Digital File 2.

**TABLE 1:** Measurements performed during the experiments. Data are the mean ± *SD* or the median (25th–75th percentile range) depending on the distribution.

	20% albumin	5% albumin	*p*‐value
Administered fluid volume (ml)	219 ± 33	877 ± 132	.001
Colloid osmotic pressure (mmHg) at baseline	25.1 ± 1.1	24.5 ± 1.2	.23
Half‐life of the PV expansion (h)	5.0 ± 2.8	6.5 ± 4.1	.29
Half‐life albumin (h)	9.5 ± 3.3	9.4 ± 4.4	.88
Plasma volume expansion (ml)			
1 h	360 (240–478)	515 (313–588)	<.02
6 h	115 (50–258)	185 (68–375)	.53
Plasma volume expansion, area under the curve (L min)			
0–1 h	18.5 (10.9–20.7)	25.1 (19.2–31.4)	<.02
1–6 h	76.1 (43.0–126.6)	100.5 (60.2–142.5)	.30^a^
Plasma sodium (mmol/L)			
0 min	141.3 ± 2.1	141.3 ± 1.4	1.0
6 h	141.9 ± 1.9	141.8 ± 1.3	.85
Plasma potassium (mmol/L)			
0 min	3.9 ± 0.3	3.8 ± 0.3	.52
6 h	3.9 ± 0.2	4.0 ± 0.5	.53
Plasma creatinine (μmol/L)			
0 min	85 ± 13	82 ± 11	<.05
6 h	79 ± 13	77 ± 13	.31
Urinary creatinine (mmol/L)			
0 min	15 ± 6^b^	14 ± 8^b^	.57
1 h	11 ± 8^b^	7 ± 6^b^	<.05
6 h	9 ± 6^b^	6 ± 2^b^	.08
Blood platelets (10^9^/L)			
0 min	210 ± 42	207 ± 39	.43
6 h	202 ± 37	199 ± 43	.68
Urinary excretion (ml)			
0–1 h	123 (81–227)^c^	271 (156–416)^d^	<.04
0–6 h	547 (316–780)	687 (626–1080)	.24

^a^ The mean values differed 14% between the groups.

^b^ Two participants excluded due to missing values at 1 h.

^c^ Three participants voided at 1.5 h.

^d^ One participant voided at 1.5 h.

The baseline MR‐proANP was 43 ± 12 and 41 ± 14 pmol/L for the 20% and 5% albumin infusions, respectively. By 30 min after the infusions ended, the MR‐proANP had increased to 54 ± 19 and 52 ± 21 pmol/L, respectively. The overall change was statistically significant (*p* < .001).

**FIGURE 1:**
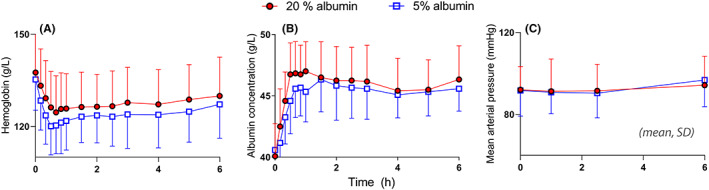
Raw data. The blood hemoglobin (A) and albumin concentrations (B), and the mean arterial pressure (C) during the trial period.

The crude blood Hb and plasma albumin concentrations are shown in Figure 1A,B. The mean arterial pressure did not change during the study (Figure 1C). The heart rate had decreased slightly at 150 min (to 59 ± 7 bpm; *p* < .02), but there were no statistically significant differences between the fluids. The mean erythrocyte corpuscular volume decreased by 0.3% during both series of experiments.

### 3.2 Fluid volumes

**FIGURE 2:**
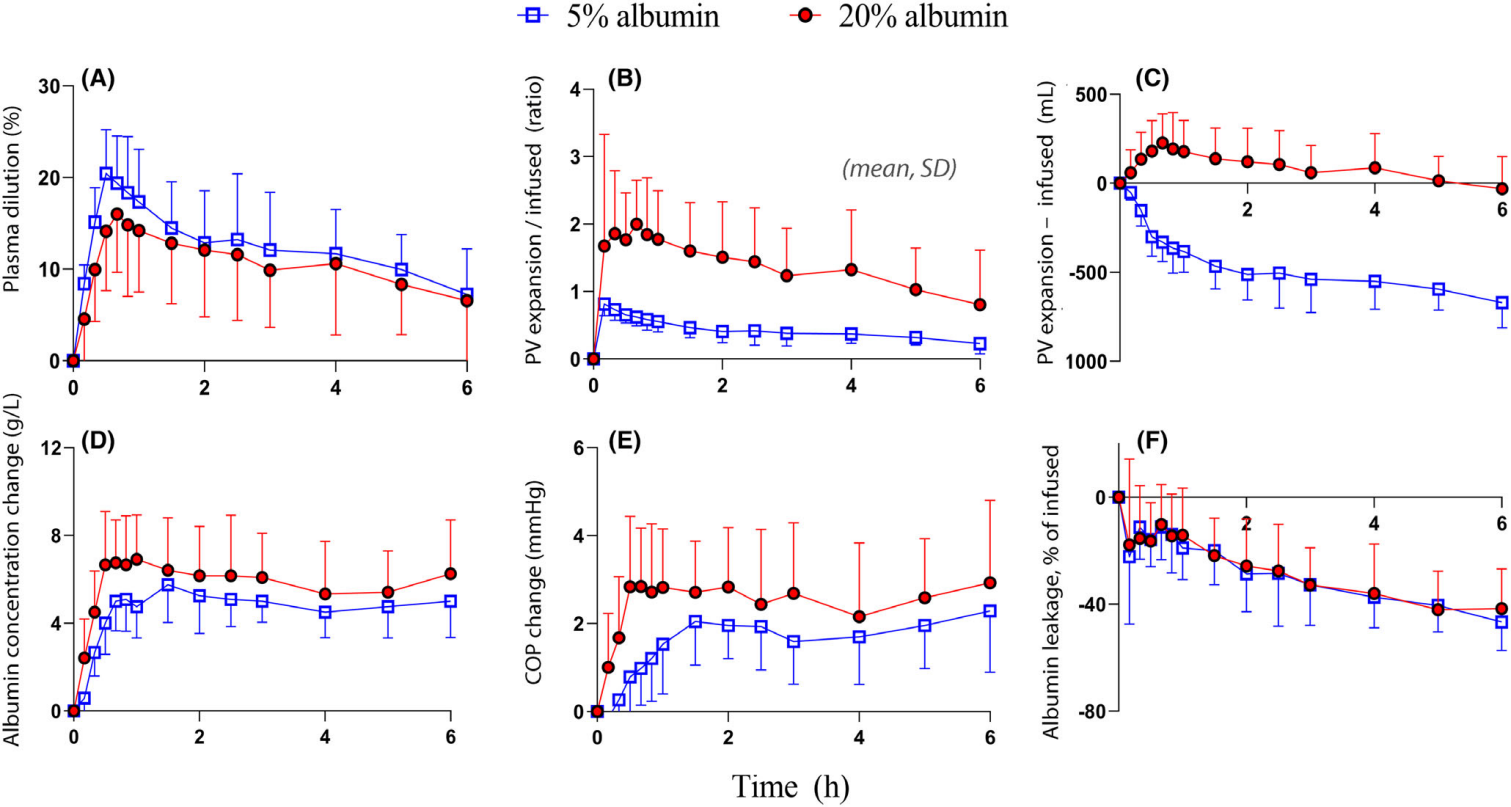
Mass balance. (A) Plasma dilution, which equals the expansion of the plasma volume in per cent. (B) Plasma volume expansion divided by the infused volume. (C) Plasma volume expansion minus the infused volume. (D) Change of plasma albumin concentration. (E) Change of the plasma colloid osmotic pressure. (F) Capillary leakage of albumin expressed as the percentage of the infused amount of albumin.

The greatest PV expansion during the 20% albumin infusion experiment was 16.0 ± 6.4% and occurred 10 min after the infusion ended. The corresponding maximum value for the 5% albumin infusion was 19.4 ± 5.2% and was recorded at the end of the infusion (*p* < .03; Figure 2A).

The baseline PV obtained by anthropometry was 2.9 ± 0.5 L. As PV expansion was taken as the product of the baseline PV and the plasma dilution, the 20% albumin expanded the PV by a maximum of 200 ± 66% of the infused fluid volume, while the 5% infusion expanded the PV by 66 ± 12% of the infused volume (*p* < .001; Figure 2B). Hence, the 20% albumin was by far the most effective PV expander (Figure 2C). The area under the curve showed that 5% albumin still expanded the PV by 35% more than 20% albumin during the first hour (*p* < .02), but the difference later lost statistical significance.

The cumulative urine output at 6 h was 548 (316–780) ml and 687 (626–1080) ml after the 20% and 5% albumin experiments, respectively (*p* = .24). The excreted urine represented 2.8 ± 1.6 and 1.0 ± 0.5 times the infused fluid volumes (*p* < .01).

### 3.3 Albumin

The plasma albumin concentration was 40.1 ± 2.6 g l^−1^ at baseline prior to the 20% albumin infusion and 40.6 ± 2.4 g l^−1^ prior to the 5% albumin infusion. These concentrations increased by 6.7 ± 2.4 g l^−1^ and 4.0 ± 1.4 g l^−1^, respectively, during the infusions (*p* < 0.01; Figure 2D).

The plasma COP was 25.1 ± 1.1 mmHg prior to the 20% albumin infusion and 24.5 ± 1.2 mmHg prior to the 5% albumin infusion. The increase in COP was 2.8 ± 1.6 mmHg at the end of the 20% infusion and 0.8 ± 1.0 mmHg after the 5% albumin infusion (*p* < .001; Figure 2E).

The dilution‐corrected plasma albumin values usually attained a virtual steady state during the first hour after the infusions ended. Therefore, the calculation of the half‐life of the excess intravascular albumin was not initiated until an apparent first‐order elimination function had been firmly established. The PV expansion did not show such steady state, and calculations could usually begin when the infusion had just ended.

So obtained, the half‐life of the excess albumin in the plasma and the PV expansion did not differ significantly between the study groups (Table 1), but the PV decreased faster than albumin (*p* < .001).

At 6 h, the capillary leakage of albumin was 42 ± 15% of the infused amount after the 20% albumin infusion and 47 ± 11% after the 5% infusion (*p* < .28; Figure 2F).

### 3.4 Oncotic pressure of the infused fluids

Duplicate measurements of 3 fresh units of 5% albumin showed a mean of 19.1 ± 0.2 mmHg. Eight samples of 20% albumin diluted by 1:4 (i.e., to 5%) yielded 19.4 ± 0.3 mmHg. One extreme outlier was deleted; no other pair differed by more than 0.1 mmHg. Three units of 20% albumin diluted 1:2 (i.e., to 10%) showed 55.2 ± 0.6 mmHg. The range of operation of the oncometer did not allow measurement of the oncotic pressure of 20% albumin, but log‐linear regression based on the other data yielded 160 mmHg.

### 3.5 Kinetic analysis

**FIGURE 3:**
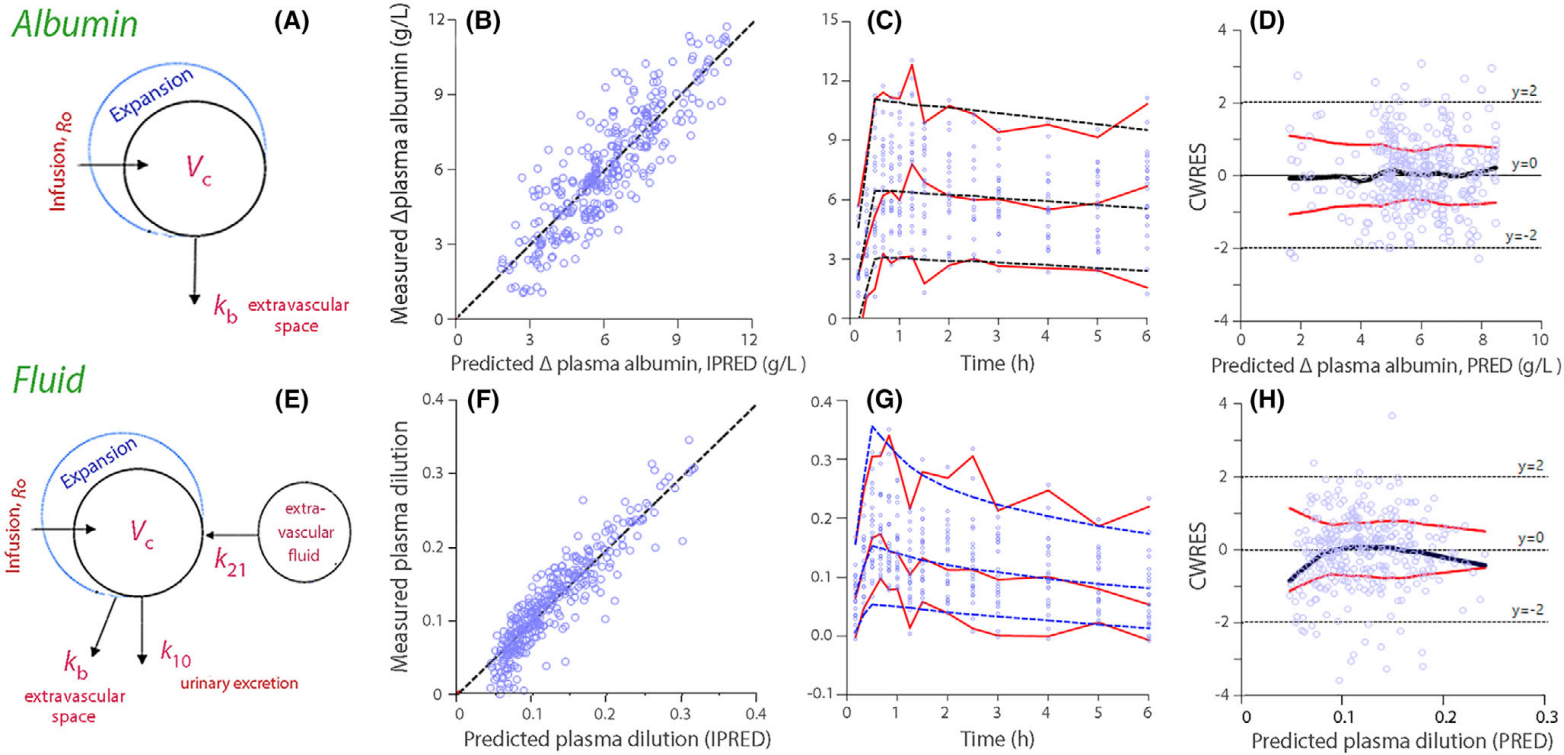
Kinetic models, goodness‐of‐fit, and performance plots. (A) Model used to analyze the kinetics of exogenous albumin. (B) Individually predicted versus measured rise in dilution‐corrected plasma albumin. Limited and evenly distributed scatter around the red line of unity represents a good fit of the model to the observed data. (C) Predictive checks for the dilution‐corrected plasma albumin. Close agreement between 5%, 50%, and 95% confidence limits based on 1000 simulations (hatched lines) and the similar limits for the observed data (solid lines) shows good model performance. (D) Conditional weighted residuals (CWRES) for the basic albumin model (without covariates).^16^ A good specification of the model yields an even distribution of residuals around zero (the straight line at *y* = 0) for gradually increasing elevations of the plasma albumin concentration. The thick black line is the logically estimated smoothing line (LOESS) and the surrounding red lines the smoothing fits to the absolute residuals. *y* = ± 2 represent ±2 standard deviations of the residuals. (E–F) Same plots as the upper row, but for the analysis of fluid volume kinetics. For all subplots: Blue circles show the individual data from all 24 experiments. Covariates are included in the performance plots except in Plots D and H.

The model used for the analysis of exogenous albumin is illustrated in Figure 3A, goodness‐of‐fit in Figure 3B, and performance measures in Figure 3C,D.

The same standard plots from the analysis of infused fluid volume are shown in Figure 3E–H. All model parameters are given in Table 2.

**TABLE 2:** Population kinetic parameters for albumin and the infused fluid volume in the final model. Shown are the typical values (tv) for the fixed parameters in the group, followed by individual‐specific covariates.

Kinetic parameter	Covariate	Covariate model	Best estimate	95% CI	CV%	−2 LL
Albumin kinetics						
tv*V*_c_ (L)			6.81	5.99–7.61	6.1	
tv*k*_b_ (10^−4^ min^−1^)			3.17	0.56–5.79	41.8	1195
*V*_c_	Albumin baseline	Power	2.25	0.22–4.28	45.9	1167
*k*_b_	MR‐proANP	Power	2.23	0.76–3.70	33.4	1157
*V*_c_	Body weight	Power	1.49	0.77–2.21	24.5	1146
Fluid kinetics						
tv*V*_c_ (L)			4.93	3.92–5.90	10.3	
tv*k*_10_ (10^−3^ min^−1^)			3.41	2.39–4.42	15.2	
tv*k*_21_ (10^−3^ min^−1^)			1.55	1.08–2.00	24.0	
tv*k*_b_ (10^−3^ min^−1^)			17.5	9.9–25.1	22.0	−963
*k*_21_ (10^−3^)	COP fluid – patient	Linear	5.24	2.04–8.44	31.1	−995
*k*_10_	Body mass index	Power	−3.84	−6.08 to −1.59	−29.8	−1003
*k*_10_	U‐creatinine, baseline	Power	−0.49	−0.73 to −0.24	−25.6	−1015

*Note*: *V*_c_ = conversion factor between mass and plasma concentration. Rate constants describing flows: *k*_b_ = capillary leakage; *k*_10_ = urinary excretion; *k*_21_ = absorption of extravascular fluid; tv = typical value for the group. CI = confidence interval. CV% = coefficient of variation (inter‐individual). LL = log likelihood for the model during development. Decrease by >6.6 points = *p* < .01. MR‐proANP = lowest quartile mean 25.3; mean 47.2; highest quartile mean 71 pmol/L. Mean albumin baseline = 40.3 g/L; mean body mass index = 24.2 kg/m^2^; Urine creatinine 15.6 mmol/L. COP of the fluid during infusion *minus* patient's plasma COP = lowest quartile mean −5.5, mean 10.8, highest 133.3 mmHg.

Covariance analysis showed that the capillary leakage of albumin, as represented by the rate constant *k*_b_, was accelerated when plasma MR‐proANP was high. By contrast, MR‐proANP was only close to be a statistically significant covariate for *k*_b_ and *k*_10_ in the fluid kinetic analysis. Instead, difference in COP between the infused fluid and the plasma COP during infusion markedly increased the recruitment of extravascular fluid (increased *k*_21_). High urine creatinine at baseline and a high BMI decreased the urine flow (*k*_10_).

### 3.6 Simulations

**FIGURE 4:**
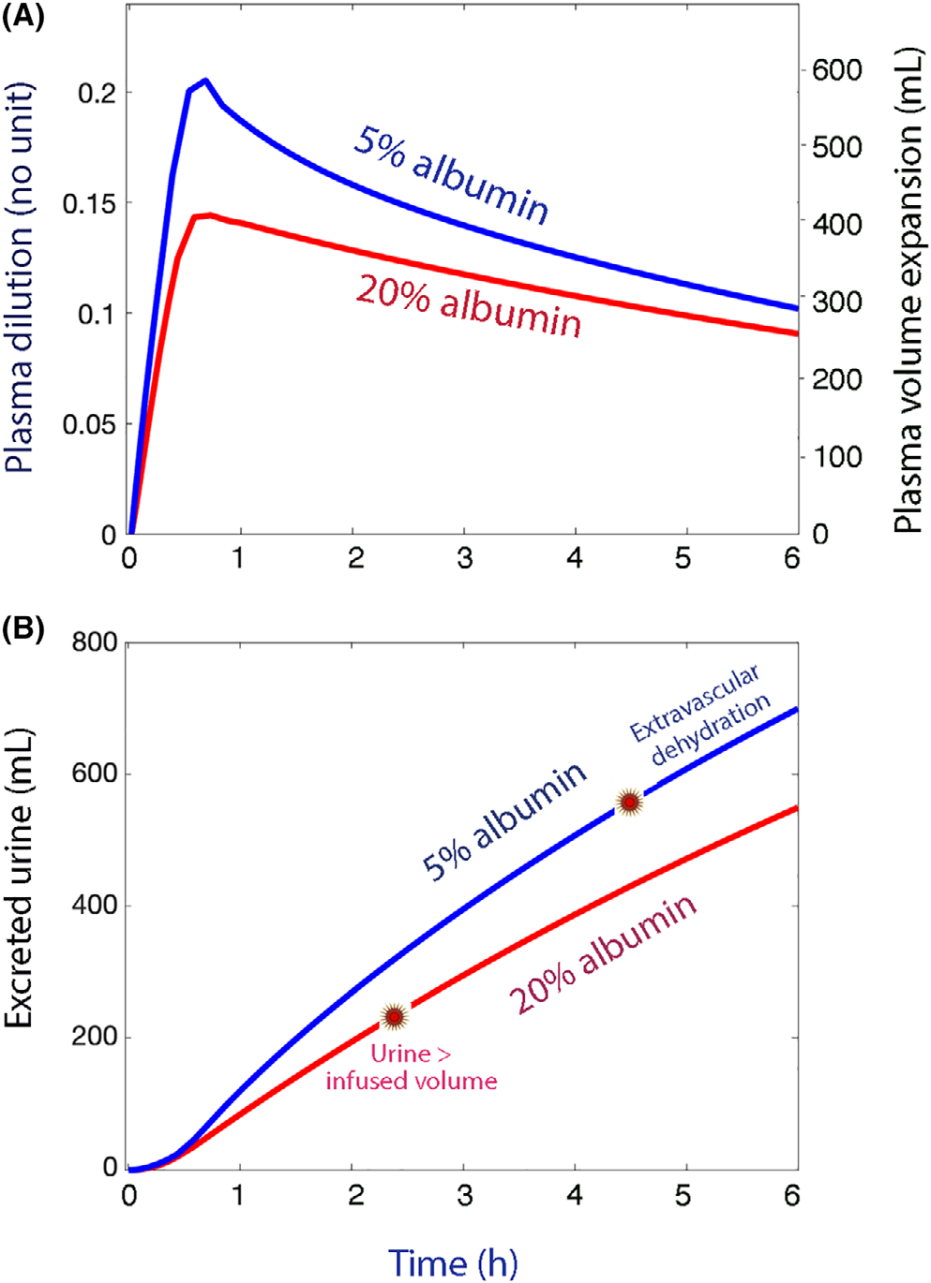
Simulation of volumes. (A) Plasma dilution (left panel) and plasma volume expansion (right panel) and (B) the excreted urine volume. Simulations were based on the best estimates for the fluid volume kinetics in Table 2. The red circle on the line for 20% albumin in subplot B marks the point in time when the excreted urine exceeds the infused fluid volume (this never occurred for 5% albumin). The red circle on the line for 5% albumin marks the point in time the dehydration of the extravascular space begins (this occurred throughout the experiment with 20% albumin). The latter period is when the infused fluid volume minus the excreted urine exceeded the plasma volume expansion.

Simulations based on the kinetic data in Table 2 confirmed that the PV expansion was slightly greater for 5% albumin, in particular during the first hour (Figure 4A) which agrees with the real data in Figure 2A. The modeled urinary excretion exceeded the infused volume of 20% albumin from 2 h and onward, while this never occurred for 5% albumin. The extravascular space lost volume throughout the experiments with 20% albumin, while this only occurred from 4.5 h and onward when 5% albumin was infused; however, the dehydration amounted only to 100 ml at 6 h (Figure 4B).

**FIGURE 5:**
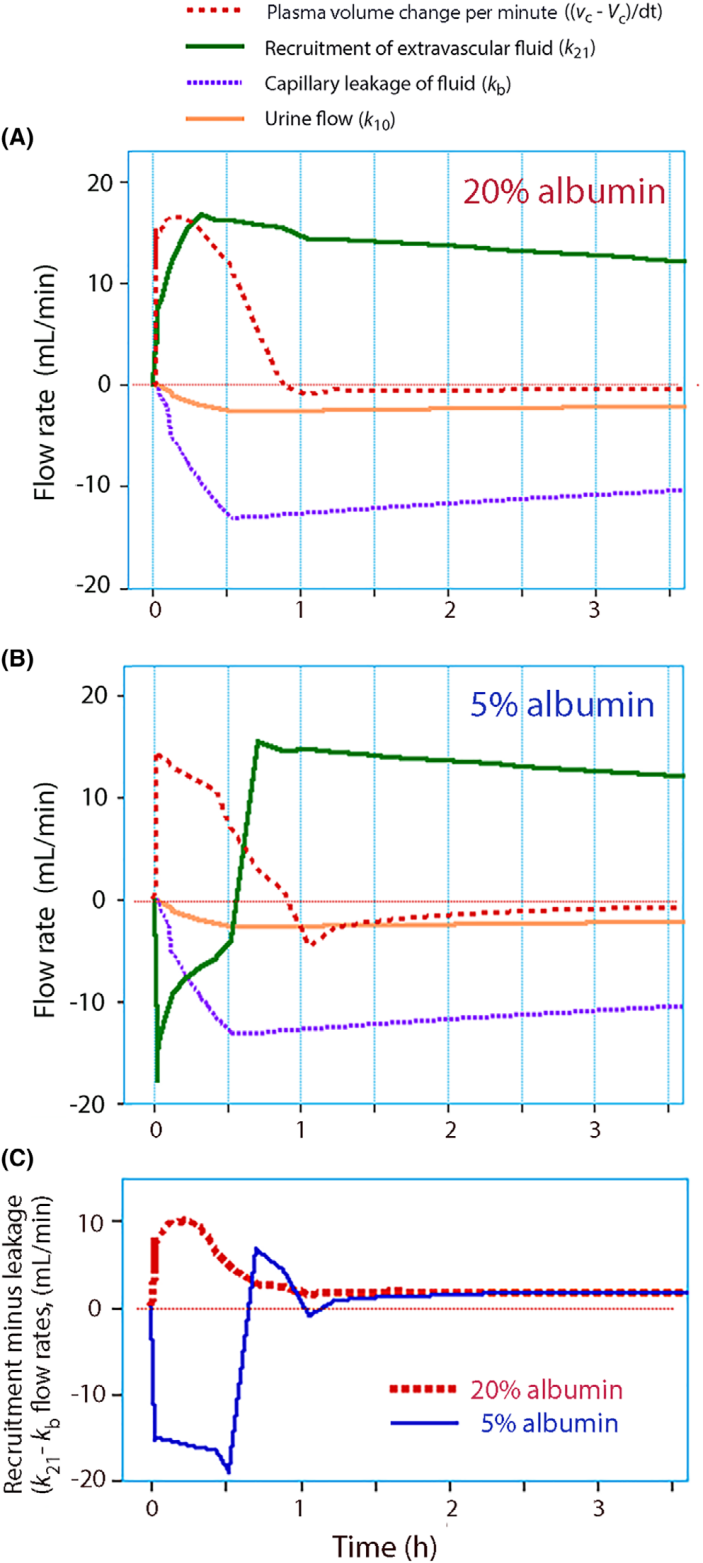
Simulation of flows. The flow rates involved in the kinetics of (A) 20% albumin and (B) 5% albumin during the first half of the experiments. Capillary leakage rate and urine flow were obtained as the product of the respective rate constant (*k*_b_ and *k*_10_) and the central volume expansion. A rate of +15 ml min^−1^ for the plasma volume means that the volume increases by 15 ml min^−1^ at that point in time. The recruitment rate of extravascular fluid was obtained as: plasma volume change – infusion rate + urine flow + capillary leak.^33^ The data were adapted to the intravascular space as the kinetic space included perivascular volume as well. (C) The difference between the recruitment of fluid from the extravascular space (*k*_21_) and the capillary leakage to the same space (*k*_b_). Balance was achieved after 1 h.

The flow rates involved in the fluid kinetics of 20% and 5% albumin are illustrated in Figure 5. The PV continued to increase for 15 min after the infusion of 20% albumin due to a lag time for recruitment of extravascular fluid (Figure 5A). Infusion of 5% albumin begun with a sharp leakage to the extravascular space, which was driven by the difference in oncotic pressure between the 5% albumin solution and the plasma (19.1 vs. 24.5 mmHg). The direction of this flow was reversed from the end of infusion and onward (Figure 5B).

The opposing flows governed by *k*_21_ and *k*_b_ did not become well balanced until after the first hour of the experiments (Figure 5C).

## 4 DISCUSSION

### 4.1 Key result

PV expansion and urinary excretion were statistically larger for 5% albumin, but only during the first hour of the experiments. Capillary leakage and recruitment of extravascular fluid soon equilibrated plasma albumin and aligned the intravascular half‐lives of exogenous albumin and the induced PV expansion. After the first hour, the kinetic profiles of 20% and 5% albumin were quite similar. The most important difference between the fluids at the end of the study was a dehydrating effect of 20% albumin.

### 4.2 PV expansion

Infusion of 3 ml kg^−1^ of 20% albumin and an equal mass of albumin as a 5% solution over 30 min in volunteers expanded the plasma volume by 19% and 16%, respectively. This difference seems small when considering that the infused volumes differed by a factor of four. The reason for the similar plasma dilution is that the water component of the 5% solution quickly leaked out of the plasma to form a transient extravascular edema.

By contrast, 20% albumin recruited extravascular fluid, albeit less than expected from the fourfold difference in albumin concentration between the two preparations. The limited recruitment is probably due to an increase in the capillary hydrostatic pressure and concentration of the extravascular COP, which would maintain a balance at a lower level. The result of the physiological adjustments is given by the area under the curve for the PV expansion, which shows that 20% albumin was three times more potent as PV expander per milliliter infused fluid than 5% albumin.

The MR‐proANP concentration increased only marginally in response to the infusions, but the fivefold variation at baseline could be of importance to the kinetics of these fluids. ANP is known to increase capillary permeability and urinary excretion.^17^

### 4.3 Urinary excretion

The urinary excretion was 25% larger with 5% albumin. Most of this difference is attributable to the first hour of the study, i.e., before intravascular volume equilibration had occurred. At 6 h, the infusions of 5% albumin were largely neutral with respect to the fluid balance (PV expansion + urine = infused volume) but about 100 ml of extravascular fluid had still been recruited to the plasma. By contrast, 20% albumin had an early dehydrating effect on the whole body (PV expansion + urine > infused volume by a factor of 4).

### 4.4 Distribution of 5% albumin

Infusion of 5% albumin seems to involve two stages of fluid flow across the capillary walls (Figure 5B).

The early extravascular distribution is probably due to the 30% lower COP of the infused fluid compared with the volunteer plasma. Increased capillary hydrostatic pressure and urinary excretion may have acted to further concentrate the intravascular proteins. For example, 5% albumin induced an increase in COP of almost 10% (Figure 2E). Moreover, our calculations suggest plasma albumin would have increased by 2.2 g l^−1^ if all 5% albumin had remained in the blood, but the measured change was twice as large (Figure 2D).

Differences were also noted between the preparations. The early rise in COP was greater than the increase in plasma albumin when 20% albumin was infused as compared with 5% albumin (cf. Figure 2D,E). The different patterns might reflect that the recruited fluid partly consisted of lymph, which contains globulins with oncotic properties.^18^ The thoracic duct lymph flow is known to increase within minutes in response to hypertonic or hyper‐oncotic fluid.^19^, ^20^, ^21^

The second stage implies that 5% albumin recruits fluid from the extravascular compartment. This phase was most evident from 1 h and onward, after which the urinary flow was greater than the reduction in the plasma volume. The flows of fluid to and from the plasma were quite similar for the two preparations after 1 h of the study (Figure 5C).

### 4.5 Albumin kinetics

Albumin molecules leaks continuously from the vascular system, but the plasma albumin concentration long remained at a pseudo‐steady state due to the concentrating effect of capillary leakage of fluid and urinary excretion. Therefore, the capillary leakage of albumin was calculated based on the product of PV dilution and plasma albumin, i.e. the product of the data shown in Figures 2A,D. The pseudo‐steady state could still not be fully accounted for in the kinetic analysis, but a more trustful estimate of the capillary leakage of albumin was obtained by mass balance applied to the data obtained after the pseudo‐steady state phase had ended. The intravascular half‐life of albumin then averaged 9.5 h, which is close to the normal leakage rate of 5% of the plasma pool per h.^22^, ^23^ A higher albumin concentration and a greater PV expansion could possibly accelerate the leakage,^24^ but plasma albumin and PV soon became similar for both fluids. No difference was detected in the capillary leakage rate of albumin between the two solutions, but the capillary leakage occurred faster in the presence of a high MR‐proANP concentration.

### 4.6 Fluid kinetics

The ability of the kinetic analysis to recreate the measured data on plasma dilution was better than for the albumin mass (cf. Figure 3F with 3B). Our parameter estimates indicate that the infusions accelerated the circulation of fluid but not of albumin. The capillary leakage of fluid occurred six times faster than for albumin. At baseline, this rate would only be twice that for albumin, as the albumin of lymph is 40% of the plasma concentration.^18^ This change in outflow was apparently well balanced by the oncotic‐driven inflow that was quantified by *k*_21_, which markedly prolonged the PV expansion.

Fluid recruitment by *k*_21_ did not occur instantly. Evidence for a lag time consists in the delay until the maximum plasma dilution was reached after infusion of 20% albumin. A comparison between the simulations shown in Figure 5A,B supports that the lag time was 10–15 min until equilibrium was reached, and even seems to be an important reason for the larger plasma volume expansion with 5% albumin. The “tail” at the lowest values in Figure 3F is also consistent with a lag time.

The negative covariance between urinary creatinine at baseline and *k*_10_ suggests that kidneys that are pre‐set to retain water continue to do so despite marked PV expansion. Similar findings have been made for crystalloid fluid in elderly men.^25^ High urine creatinine in the morning urine is a biomarker of low habitual intake of water and appears to adapt slowly to changes in diet.^15^, ^26^

### 4.7 Study group

The experiments were performed in healthy volunteers to allow a minimum of confounders due to health status and clinical issues. However, the volume kinetics of 20% albumin has been similar in several examined physiological situations. There were no differences between healthy volunteers and to postoperative and post‐burn patients with a marked inflammatory response.^8^, ^9^ The oncotic‐driven recruitment of fluid (*k*_21_) even attained an almost identical value in the present study as in edematous post‐burn patients.^27^ However, the intravascular persistence of 20% albumin in patients undergoing lengthy but minor surgery was even longer than in the other studies.^28^

The kinetic profile of 5% albumin may not be as stable as for 20% albumin between different clinical situations, although this is not well studied. A previous study of healthy volunteers showed that 5% albumin expanded the PV by 75% of the infused volume,^29^ which agrees well with the 66% found here. We hypothesize that the PV expansion would have been greater had the plasma albumin been lower because the COP of the plasma and infused fluid would then be more similar. Examples of this type of setting include hypoalbuminemia in severe disease and the plasma dilution caused by infusion of clear fluids during general anesthesia.^10^

### 4.8 Clinical implications

The present results show that 5% and 20% albumin have equivalent effects after an initial 1‐h period of body fluid redistributions that are likely to be driven by the differences in oncotic pressure between the infused fluids and the plasma. Both preparations provide long‐lasting plasma volume expansion. The clinically most important difference is a dehydrating effect of 20% albumin. Our kinetic analysis showed that MR‐proANP accelerated capillary leakage of albumin, whereas high BMI and a high urinary creatinine concentration retarded the urinary excretion.

Differences in kinetic profile are quite small between the present experiments and clinical studies using the same protoclol.^8^, ^9^, ^27^, ^28^ The similar pharmacologic profiles for 5% and 20% albumin suggest that both fluids can be used in clinical scenarios of moderate severity without concerns about altered kinetics. The long intravascular persistence is a concern, as four half‐lives are required before 95% of the volume expansion has subsided. Hence, the induced plasma volume expansion remains until the next day. Hyper‐oncotic albumin is most useful when a diuretic effect is desired. The 5% albumin was largely indifferent with respect to the fluid balance at the end of the 6‐hour period.

The results are likely to be generalizable with some exceptions. Capillary leakage might occur faster during major surgery.^23^ The diuretic responses to the fluids can be different in patients with impaired kidney function.

### 4.9 Limitations

We are not aware of any previous clinical trial with a detailed comparison between 5% and 20% albumin. Nevertheless, our study has several limitations.

The study was not fully randomized due to emigration and pregnancy among the volunteers, which also made it difficult to include the third study arm in this presentation as the control subjects differed.

Blood sampling should have been longer to sufficiently allow precise characterization of the elimination phases by using pharmacokinetic analysis. However, changes in body position and intake of food and water would probably confound such an extension, and a duration of 6 h is a very long experiment for volunteers to participate in the study. The duration of the study still covered one half‐life of the PV expansion and somewhat less for the albumin mass, a difference that might have been due to the reason that some albumin was recruited via the lymph. By contrast, the capillary leakage rate calculated by radioisotopes is usually based on collection of data during less than 1 h.

The present study is the first to apply a single model for analysis of the fluid volume kinetics of two preparations that have fundamentally different oncotic properties. The model may not be perfect, despite the good behavior shown by the goodness‐of‐fit and performance analyses. The fluid kinetic model does not describe a loop between *k*_b_ and *k*_21_ although a link between them certainly exists, just as albumin leaking out of the PV is later returned via the lymph. Kinetic analysis of crystalloid fluid is based on that extravascular fluid is returned to the plasma in proportion to the expansion of the extravascular space,^10^, ^24^ but this approach does not give meaningful parameter estimates when applied to 20% albumin. A balance between *k*_b_ and *k*_21_ develops during the experiments, but not during the first hour when oncotic forces apparently determine the fluid distribution (Figure 5C).

The infused fluids were at room temperature, which may have caused a minor reduction of the body temperature (<0.5°C) during the experiments with 5% albumin.^30^ The heat loss caused by infusing 1 L of fluid at room temperature amounts to 16 kcal, which does not change the hemodilution or COP.^31^

Correction of the data was made for blood sampling, but not for evaporation losses. Correction for evaporation losses would prolong the calculated half‐lives. However, the difference would be marginal as evaporated water is taken from the total body water, which would be in the range of 40–45 L in our volunteers.^32^

The reduction of the mean erythrocyte corpuscular volume is probably due to translocation of fluid from the cells because the urinary sodium concentration is initially lower than the sodium concentration of the infused fluid. In another series of volunteers, the recruited volume from the cells averaged 130 ml in response to 200 ml of 20% albumin.^33^

## 5 CONCLUSIONS

The results confirm that the dosing of 5% and 20% albumin should be adjusted according to the albumin content of the fluid and not to the infused fluid volume. On doing so, the kinetic profiles of the two solutions were similar, although 20% albumin solution was three times more potent as a PV expander than 5% albumin. This study did not find any reason to recommend one preparation over the other in the clinical setting. An exception would be that the dehydrating effect of 20% albumin could be preferred for the prevention and treatment of peripheral fluid overload.

## AUTHOR'S CONTRIBUTIONS

MZ contributed to study concept, patient recruitment, data collection, data analysis, and co‐writing of the manuscript. RGH contributed to study design, data analysis, kinetic analysis, and manuscript preparation.

## FUNDING INFORMATION

This work was supported by a grant from Grifols. The funding organization played no role in the design, analysis, interpretation of the data, or writing of the manuscript.

## CONFLICT OF INTEREST

RGH holds a research grant from Grifols. MZ declares that he has no conflicts of interest.

## Supporting information


Appendix S1

Appendix S2
