# Supplementary material for: Kinetics of 5% and 20% albumin: A controlled crossover trial in volunteers
Source: Acta Anaesthesiol Scand. 2022 May 13;66(7):847–58. doi: 10.1111/aas.14074 (PMC9541965; doi:10.1111/aas.14074)
Supplement: Supplementary file 1 — Appendix S1 [file AAS-66-847-s002.docx]

**Supplementary Digital File 1**

**Mass balance**

***Volume expansion.*** Calculation of the volume expansion based on mass balance assumes an even distribution of hemoglobin (Hgb) in the blood volume (BV) at each time of measurement. Expansion of the blood volume is inferred from the dilution of the measured Hgb concentration when fluid is infused intravenously.

In the present study, the BV prior to the infusion of 20% albumin (BV_0,_ in liters) was obtained by using Nadler’s formula, which uses the height (h, in meters), weight (w, in kg) and sex as follows (Nadler, Hidalgo & Bloch: *Surgery* 1962;51:224-232):

| Male: $\mathrm{BV}_{o} = 0.3669 h^{3} + 0.03219 w + 0.6041$ |
| --- |
| Female: $\mathrm{BV}_{o}= 0.3561 h^{3} + 0.03308 w + 0.1833$ |

The further approach is to calculate the total amount of Hgb in the circulation, which is corrected for dilution and estimated Hb losses (Hahn RG: *Acta Anaesthesiol Scand* 1987;31:572-578). For example, assume that Hb_mass(0)_ is the amount of Hgb in the circulation at time 0 (usually about 1 kg), BV_0_ is the estimated blood volume at baseline, and Hgb_0_ is the measured blood Hgb concentration which is repeated at a later time (t):

### Hgb_mass(0)_ = BV_0_ Hgb_0_

### BV_t_ = (Hgb_mass(0)_ – Hgb _loss(t-0)_ ) / Hgb_t_

### ∆BV_t_ = BV_t_ – BV_0_

### ∆PV_t-0_ = BV_t_ (1– Hct_t_) – BV_0_ (1– Hct_0_)

### Where Hct is the hematocrit. Hb_loss_ is obtained as the product of the Hb_0_ and the volume of the surgical bleeding if hemorrhage occurs rapidly. When the bleeding is slow, one multiplies the bled volume by the average of Hgb_0_ and Hgb_t_. In the present work, Hgb_loss_ represents sampled blood only as no hemorrhage was inflicted.

***Plasma dilution.*** In the simplest case, there is no loss of Hgb molecules during the experiment. Then:

Plasma dilution = [(Hgb_0_ / Hgb_t_) – 1] / (1 – Hct_0_)

Here, Hct_t_ should *not* be applied. However, as nearly all experiments include blood sampling and/or hemorrhage, the usual way to calculate plasma dilution is to apply the mass balance calculations of blood and plasma volume changes described above. Then:

Plasma dilution = [ (PV_t_ – PV_0_ ) / PV_0_ ] = [ (PV_t_ / PV_0_ ) – 1].

Note that the baseline PV is placed in the denominator here while the later measured Hgb_t_ is placed in the denominator if plasma dilution is derived directly from the change in blood hemoglobin concentration.

The use of volume ratio instead of the Hgb ratio has the downside that the BV_0_ must be assumed. However, erroneous estimates of BV_0_ ( ± 0.5 L) have little consequence for the plasma dilution as its only purpose is to correct the calculations for “false” dilution that occurs due to loss of Hgb molecules, which serve as endogenous biomarker.

How plasma dilution can be derived from the equations above is shown in the online supplement to another publication: Gunnström M, Zdolsek J, Hahn RG. doi: 10.1213/ANE.0000000000005802.

***Albumin leakage.*** The capillary leakage of albumin was based on the assumption that albumin is evenly distributed in the plasma volume (PV), which is given by the product of BV and (1**–** Hct). In the equations below, the measured plasma albumin concentration is P-Alb_0_ at baseline and P-Alb_t_ at a later time t:

The capillary leakage of albumin between times 0 and t is then calculated from the change in intravascular albumin mass with correction for the infused mass:

Albumin leak = Infused albumin + (PV_o_ P-Alb_o_) – (PV_t_ P-Alb_t_)

***Half-lives.*** The intravascular half-life (T_1/2_) of the infused amounts of albumin and fluid were calculated from a simple wash-out equation:

dX/dt = X_0_ e ^-^*^k^* ^(t )^ ,

where X_o_ is the excess intravascular mass of albumin or excess fluid volume (i.e., above baseline), *k* is the elimination rate constant, and ln 2 is the natural logarithm of 2 (which is 0.693).

**Volume kinetics**

Volume kinetic calculations do not assume that Hgb is evenly distributed in the BV at each time of measurement. The Hgb decrease in response to infusion of fluid rather indicates the size of a functional central fluid space (*V*_c_) in which infused fluid distributes very quickly. This volume can be almost identical to the physiological PV but *V*_c_ can also be larger in case the fluid rapidly distributes outside the PV.

Benefits of using volume kinetic analysis include that flows in and out of *V*_c_ may be estimated and simulated. Dynamic events can be studied, which is difficult with radioactive tracer methods. The kinetic model can also be varied depending on the type of studied fluid.

In the present study, volume kinetic analysis was applied on both the infused excess mass of albumin and the infused fluid volume.

***Albumin mass.*** The kinetics of administered albumin mass was calculated with account taken for the fact that P-Alb is artificially low due to the PV expansion.

The change in the "corrected" albumin concentration above baseline (C_alb excess_) from time o to time t was given by:

dC_alb excess_ /dt = *R*_o_ – *k*_b_ [ (PV_t_ – PV_o_) (P-Alb_t_ – P-Alb_o_) ]

where *R*_o_ is the rate of administration of albumin and *k*_b_ is the rate constant describing the loss (capillary leakage) of intravascular albumin mass.

There are two fixed output parameters: the volume of distribution of the infused albumin molecules (*V*_c_) and the elimination rate constant *k*_b_. The T_1/2_ of the albumin excess albumin can be obtained here, too, by taking ln 2 (=0.693) / *k*_b_.

The two fixed parameters in the kinetic analysis of albumin (*V*_c_ and *k*_b_) were estimated simultaneously for all 24 experiments using the First Order Conditional Estimation Extended Least-Squares (FOCE ELS) search routine in the Phoenix software for nonlinear mixed effects (NLME), version 8.2 (Pharsight, St. Louis, MO), and the additive model for the within-subject variability.

***Fluid volume.*** How to analyze the kinetics of the infused fluid volume in 20% albumin has recently been described (Hahn RG, Zdolsek M, Hasselgren E, Zdolsek J, Björne H. *Br J Clin Pharmacol* 2019; 85: 1303–1311). Here, the input data consists of frequently measured plasma dilution and the urinary excretion

The kinetic model for 20% albumin implies that fluid is infused by a rate *R*_o_ into the central body fluid space *V*_c_, which is then expanded to *v*_c_. Fluid is recruited from the interstitial fluid space volume (IFV; assumed to be 15% of the body weight) to the plasma, which is (probably) due to the increase in oncotic pressure resulting from the infused excess of albumin molecules. The rate of the absorption is governed by a rate constant *k*_21_.

Elimination of fluid occurs by urinary excretion and capillary leakage at rates proportional to the expansion of *V*_c_ by the rate constants *k*_10_ and *k*_b_. The finally developed ‘base model’ was expressed by the following equations, where *u* denotes the measured urinary excretion (the term *k*_21_ IFV applies only when the infusion has started):

d*v*_c_ /dt = *R*_o_ – *k*_b_ (*v*_c_ – *V*_c_) – *k*_10_ (*v*_c_ – *V*_c_) + *k*_21_ IFV

dIFV/dt = – *k*_21_ IFV_o_

d*u*/dt = *k*_10_ (*v*_c_ – *V*_c_)

Please note that *k*_21_ is only operational when 20% albumin is or has been infused. The four fixed parameters in the fluid model base model (*V*_c_, *k*_10_, *k*_b_, and *k*_21_) were estimated for all 24 experiments simultaneously by using the Phoenix NLME software, which means that both 20% and 5% albumin were analyzed using the same kinetic model. The only effect on assuming a different IFV is that *k*_21_ will obtain a slightly different value.

***Covariate analysis.*** The kinetic analyses were made using software for mixed effects which allows refinement of the models by introducing individual-specific *covariates* such as age, gender, and body weight. The use of such analyses is a standard tool for evaluating and recommending suitable dosing regimens for drugs based on personal characteristics.

The principles for this analytical approach are explained in detail in: Owen JS, Fiedler-Kelly J. *Introduction to population pharmacokinetic/pharmacodynamic analysis with nonlinear mixed effects models.* Hoboken: Wiley & Sons, 2014.

The search for covariates to the fixed parameters that could reduce the residual error was guided by plots of random effects (eta:s). The most promising candidate parameters were then tested, one by one, by adding them to the model. In the present work, we started with 15 variables that were of potential interest (maximal number in the analysis program, Phoenix NMLE) of which 10 were sufficiently interesting to be evaluated in detail. These variables were thought to have potential for influencing the kinetics of the albumin or fluid kinetics.

Testing was made in sequence to all parameters in the base model. The covariate was accepted for inclusion if it significantly improved the goodness-of-fit for the model; a reduction of -2 LL (LL = log likelihood) by >3.8 points represents *P*< 0.05 and >6.6 points represents *P*< 0.01. Furthermore, zero was not allowed to be within the 95% confidence interval of the covariate, and the between-subject variability had to be < 50%. The fixed parameters in the base model and the statistically significant covariates were always estimated simultaneously using the Phoenix software.

Simple explanatory texts about volume kinetics and essential findings are given in: Hahn RG. *Acta Anaesthesiol Scand* 2020;64:570–578 and Choi B-M. *Korean J Anesthesiol* 2021;74:204-217.
